# Triptan use in elderly over 65 years and the risk of hospitalization for serious vascular events

**DOI:** 10.1186/s10194-024-01770-x

**Published:** 2024-04-26

**Authors:** Phuong Thao Tran, Maryse Lapeyre-Mestre, Baricault Berangere, Michel Lanteri-Minet, Aurore Palmaro, Anne Donnet, Joëlle Micallef

**Affiliations:** 1 Service de Pharmacologie Médicale et Clinique, Université de Toulouse, CHU de Toulouse, Toulouse, France; 2 College of Pharmacy, Seoul National University, Seoul, South Korea; 3 PEPSS “Pharmacologie En Population cohorteS et biobanqueS”, Centre d’Investigation Clinique Inserm (CIC 1436), Université de Toulouse, Toulouse, France; 4 Neuro-Dol Inserm U1107, Université Clermont Auvergne, Clermont-Ferrand, France; 5 Département d’évaluation et de traitement de la douleur, CHU de Nice, FHU InovPain Université Côte Azur, Nice, France; 6 Centre d’Evaluation et de Traitement de la douleur, FHU InovPain Pôle Neurosciences Cliniques, APHM, Marseille, France; 7 service de pharmacologie clinique & pharmacosurveillance, centre régional de pharmacovigilance, Aix-Marseille université, Inserm, UMR 1106, Assistance publique-Hôpitaux de Marseille, Hopital Sainte Marguerite 270, boulevard sainte Marguerite, Marseille, 13009 France

**Keywords:** Triptan, Elderly, Stroke, Myocardial infarction

## Abstract

**Background:**

Several studies have focused on the use of triptan and the risk of acute vascular events but the existence of such association is still debated and has never been quantified in patients over 65 years. To assess whether triptan use among older is associated with an increased risk of hospitalization for acute vascular events.

**Methods:**

A propensity score-matched cohort study was designed using the French national health insurance database linked to hospital stays. Patients aged ≥ 65 years, newly treated by triptans between 2011 and 2014, were included… The primary event was hospitalization for an acute ischemic vascular event within de 90 days following triptan initiation. Association with triptan exposure was investigated through cox regression model, considering exposure at inclusion, and with exposure as a time-varying variable A case-crossover (CCO) and a self-controlled case series (SCCS) analyses were also conducted to address potential residual confounding.

**Results:**

The cohort included 24, 774 triptan users and 99 096 propensity matched controls (mean (SD) age: 71 years (5.9), 74% of women). Within 90 days after cohort entry, 163 events were observed in the triptan group, and 523 in the control group (0.66% vs. 0.53%, adjusted hazard ratio (aHR) _exposed/not exposed_ 1.25 95%CI [1.05–1.49]; aHR _time−varying_ 8.74 [5.21–14.66]). The association was significant (CCO) for all events (adjusted odds ratio (aOR1.63 [1.22–2.19]) with a more consistent association with cerebral events (aOR 2.14 [1.26–3.63]). The relative incidence (RI) for all events was 2.13 [1.76–2.58] in the SCCS, for cardiac (RI: 1.67 [1.23–2.27]) and for cerebral events (RI: 3.20, [2.30–4.45]).

**Conclusion:**

The incidence of acute vascular events was low among triptan users. We found that triptan use among older may be associated with a low increased risk for acute vascular events, which may be more marked for cerebral events such as stroke, than for cardiac events.

**Supplementary Information:**

The online version contains supplementary material available at 10.1186/s10194-024-01770-x.

## Introduction

Sumatriptan and the other second-generation serotonin 5HT1B/1D-receptor agonists (triptans) have improved the quality of life of migrainers by providing a higher degree of efficacy and a more favorable safety profile, in comparison with ergotamine. However, due to their 5HT1 agonist activity, triptans can also cause coronary, cerebrovascular and peripheral vasoconstriction possibly leading to serious outcomes such as myocardial infarction, ischemic stroke and ischemic colitis, mostly in patients with cardiovascular disease or risk factors [1–3]. Since the first approval of sumatriptan for the treatment of migraine attack, this cardiovascular risk is still debated. The conclusions of the Triptan Cardiovascular Safety Expert Panel, a multidisciplinary group of experts in neurology, primary care, cardiology, pharmacology, women’s health, and epidemiology, were very reassuring [4]. The incidence of acute vascular events with triptans in both clinical trials and clinical practice appears to be extremely low, and the cardiovascular risk-benefit profile of triptans favours their use in the absence of contraindications.

Some studies have assessed the relationship between triptans and cardiovascular events [2, 5–9]. They concluded either in the absence of risk or a moderately increased risk of vascular events, but none of these studies considered older age. Indeed, triptans are labelled only for patients between 18 and 65 years. Prescription in older patients is not recommended according to the summary of product characteristics, as safety and efficacy has not yet been established. They are then contraindicated in case of past history of ischemic cardiovascular conditions (myocardial infarction, coronary vasospasm), peripheral arterial pathology, arterial hypertension or stroke.

In spite of the contraindication and the higher prevalence of these medical conditions in people over 65, triptan use in patients over 65 is quite common, both in France and other countries [2, 10–13]. Observational studies in general population have estimated that patients older than 65 may represent 5 to 8% of all triptan users [2, 10–13]. Nevertheless, to our knowledge, no study has investigated specifically cardiovascular safety among older patients exposed to triptan. The aim of the study was to assess whether triptan use among patients older than 65 is associated with an increased risk of hospitalization for acute vascular events. We implemented a propensity score-matched cohort, with complementary analysis using case-crossovers analysis and self-controlled cas series resulting in the control time independent confounding factors.

## Methods

### Data source

This study was based on data from the SNDS (Systeme National de Données de Santé) [14, 15], the national electronic health care database linked to vital statistics in France. The SNDS includes health care data for more than 67 million individuals living in France, from birth (or immigration) to death, and covered by the national health insurance system, which is mandatory in France. The SNDS contains individualized, anonymized data on demographics (sex, year of birth, date of death if relevant); on health status through long term disease (LTD) status resulting in full insurance coverage for the patient with at least one LTD; all reimbursed outpatient healthcare encounters (visits, medical procedures, lab tests, drugs, medical devices); all hospital procedures and discharge diagnoses both for public or private hospitals (with main diagnosis, related diagnosis, and as many associated diagnoses as necessary for one hospital discharge summary). Diagnoses identified in LTD and in hospital discharge summaries are coded according the 10th revision of the International Classification of Diseases (ICD-10). Information available for any prescribed and reimbursed drug is drug name, dosage, form, quantity and dates of prescription and dispensing in community pharmacies [14–16]. The SNDS has been extensively used in epidemiology and pharmacoepidemiology, in particular to assess cardiovascular disease management and outcomes [17–20].

For the purpose of this study, data were extracted from the SNDS in January 2017 after ethics and regulatory agreements, and covered the period from January 1st 2011 to December 31st 2014. Available information was demographics (including vital status), reimbursed drugs, LTD and hospitalisation data.

#### Patient selection

Subjects over 65 years old at the time of a first dispensing of any triptan between July 1st 2011 and June 30 2014 were selected and defined as new users of triptan (if nodispensing of any triptan had been given in the 6 months preceding the date of the firstdispensing identified in the study period). Subjects with adispensing of subcutaneous sumatriptan for cluster headache as well subjects concurrently prescribed ergot alcaloides and triptans were excluded. Each incident user of triptan was matched with four unexposed controls, on age, sex, and area of residence. The exclusion of the 6 first months in 2011 insured to include only incident users, as well the exclusion of the 6 last months in 2014 allowed to have at least 6 months of follow up for all subjects included in the cohort.

### Study design

The data analysis was performed in 2 steps. First, from the exposed-unexposed cohort extracted from the SNDS, we implemented a propensity score-matched cohort study comparing the incidence of cardiovascular outcomes and death according to triptan exposure. Second, in order to address potential residual confounding, we conducted complementary analyses where subjects were their own controls which allows self-adjusting over a short period for individual time invariant characteristics that are not recorded in medico-administrative healthcare databases (such as diagnostic of migraine or cigarette smoking, alcohol consumption, overweight or obesity,…): a case-crossover (CCO) analysis and a self-controlled case series (SCCS) [21–24].

### Primary analysis: propensity score matched cohort study

#### Selection of triptan users and matched controls

The characteristics of the initial exposed-unexposed cohort identified in the SNDS were unbalanced, unexposed controls presenting more comorbidities and health care consumption than incident triptan users. A propensity score was computed trough a logistic regression model including baseline covariates available before cohort entry (index date being the date of the firstdispensing of triptan for triptan users and for the 4 matched-controls). These variables were sex, age, Charlson’s comorbidies score (CCS) estimated from ICD-10 codes identified in hospitalisation diagnoses, LTD, and/or drug dispensing [25–28] comorbidities known as risk factors of cardiovascular events (hypertension, cardiovascular disease, dyslipidemia and diabetes), number of medical visits and number of hospitalisations within the six months before index date. The list of codes used for identifying these covariates is provided in supplementary Table 1.

We performed a nearest neighbor matching with a caliper of width equal to 0.2 of the standard deviation. Distances before and after propensity score matching between triptan users and controls were investigated through Cohen’s d computation. Subjects in the PS matched cohort were followed for up to 90 days from the index date until predefined outcome.

#### Exposure

Triptan exposure was assessed through data available in the SNDS and included triptans available in France: sumatriptan (except subcutaneous sumatriptan for cluster headache), naratriptan, zolmitriptan, rizatriptan, almotriptan, eletriptan, frovatriptan. Quantities provided for each triptan at each dispensing were converted in defined daily dose (DDD) using the ATC/DDD index of the WHO Collaborating Centre for Drug Statistics Methodology [29]. According to this methodology, one DDD for a given drug is the assumed average maintenance dose per day for this drug used for its main indication in adults. Because data available in the SNDS do not allow to know the exact exposure in patients, we considered that they were exposed from the day of dispensing (index date) until the end of treatment period corresponding to the number of days of supply (number of provided DDDs, days of treatment at the assumed average of daily dose). For example, the DDD value is 2.5 mg for frovatriptan, naratriptan and zolmitriptan, or 40 mg for eletriptan (per os).

#### Outcomes

The main study outcome was acute ischemic vascular events, defined by an hospital admission within the 90 days following index date with a main diagnosis of a list ICD-10 codes, having a priori the best positive predictive values [17–20, 24, 30] for these events (supplementary Table 2). The secondary outcomes were death of any cause within the 90 days following index date and death occurring in the 30 days after hospital admission for an ischemic cardiovascular event.

### Secondary analyses

Because the residual confounding in the exposed-unexposed cohort study, we added complementary analyses based on self-controlled designs. In that designs, only individuals with the event of interest are considered, which then act as their own control (i.e., they consist in within-patient comparison between different periods of time). Their main advantage is that time-invariant confounders that act multiplicatively on the event rates are inherently controlled for. These designs include the case-crossover (CCO) design and the self-controlled case-series (SCCS) and were developed in the early 90’s to study the short term effect of transient-exposures and abrupt onset events ^22^. Indeed, because of the self-matched design, the risk estimation includes only data for patients who switch their exposure status over time (i.e., from exposed to unexposed, or vice versa). Because exposure to triptans is occasional and study outcomes are acute by definition, these methods are appropriate to insure the robustness of results from the exposed-non exposed cohort.

The CCO designs were initially developed by Maclure in 1991 [21], with initial applications being the study of the triggering factors of myocardial infarction, or road injuries. They were then used in pharmacoepidemiology to study the association between drug exposure and the occurrence of an event.

All subjects with the event of interest (cases) are identified and included in the study. The exposure is collected over the so-called “risk period” immediately preceding the event, then over one or more previous periods so-called “control periods”. If an association exists between the exposure and the event, more frequent exposure should be observed during risk periods compared to control periods. This association is estimated through the calculation of a CCO odds-ratio (OR): the exposure rate in the case periods is reported to the exposure rate in the control periods in the same subject. Only the discordant pairs of subjects are considered, since patients whose exposure status does not vary over risk and control periods do not contribute to the calculation.

SCCS designs were initially introduced to investigate the potential relationships between Measles Mumps Rubella vaccination and aseptic meningitis, but have been widely used to study other vaccine risks, and extended to other adverse drug events [23–24].

As in the CCO design, only cases are included, but in contrast to the CCO, the entire exposure history inside a given time window is retrieved, not just exposure attributes of selected dates or periods. Other important features of SCCS are that the exposure history occurring after the event is included in the estimates. The event rate is compared between person-times at risk (risk periods with exposure) and person-times not at risk (baseline periods) in the same individual. Unlike CCO studies for which periods are defined a priori, SCCS expand the self-controlled method to periods with different sizes, and use all the information available during the observation period [23–24]. As with CCO studies, case selection must be done without knowledge of the potential exposure. The risk function for one individual is considered as depending on age and on his/her exposure at each time.

For the self-controlled analyses, the observation period to select cases began from 01/07/2011 (or at age 65) and ended on 31/12/2014 (or eventually the day of death). In this period, subjects in the triptan exposed cohort and hospitalized at least once for an ischemic event were identified. Due to the lack of a common formula for the population size (for CCO), we included as much as possible eligible subjects. For SCCS, with a level of significance at 5% and a power at 80%, and for an expected relative risk of 1.3 and a risk period/baseline periods ratio of 0.1, the required population was 1192 subjects.

### Case-Cross Over (CCO)

#### Definition of index date and risk periods

**Fig. 1:**
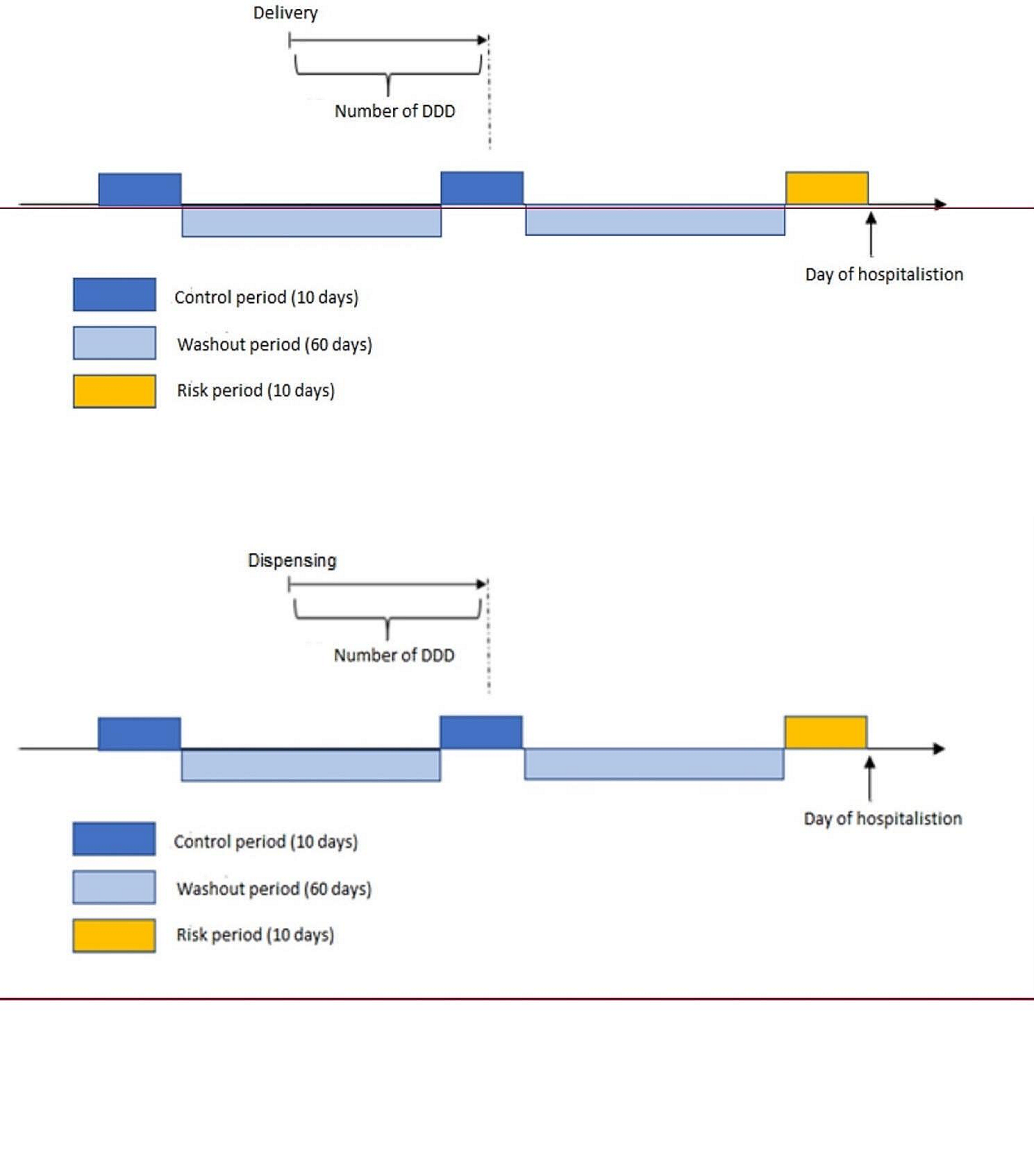
Study design of the case cross over analysis. All subjects with an event of interest (first hospitalisation for an ischemic event) and exposed to triptans before the event were selected (from the exposed cohort). They were observed from the beginning of the cohort (01/07/2011) or when aged 65, to the end (31/12/2014.). Exposure to triptans(beginning on day of dispensing and covering a number of days corresponding to the number of supplied DDD), was compared between risk period (yellow) and control periods (dark blue). Exposure during washout periods (light blue) was neutral. Duration of exposure was modified by adding 7, 14 and 28 days in sensitivity analyses. The risk period for an ischemic event was defined as 10 days before the day of hospital admission

All subjects with an event of interest (hospitalization for an ischemic event) after inclusion were identified. Triptan exposure immediately before the event (risk period) was compared with triptan exposure in two previous and separated periods (control periods). The reference event was the first event observed. The index date was the day of hospitalisation (named T0) and the risk period was therefore the 10-day period preceding the index date. The control periods were two previous 10-day periods, separated by washout periods of 60 days, to ensure that the autocorrelation do not affect the results (Fig. 1).

Triptan exposure was defined by at least one day of triptan (one DDD) in risk or control periods. No exposure was defined by no overlap with the considered periods.

The analysis was performed by type of events (all ischemic vascular events, ischemic cardiac events, and ischemic cerebral events).

### Self controlled case series (SCCS)

**Fig. 2:**
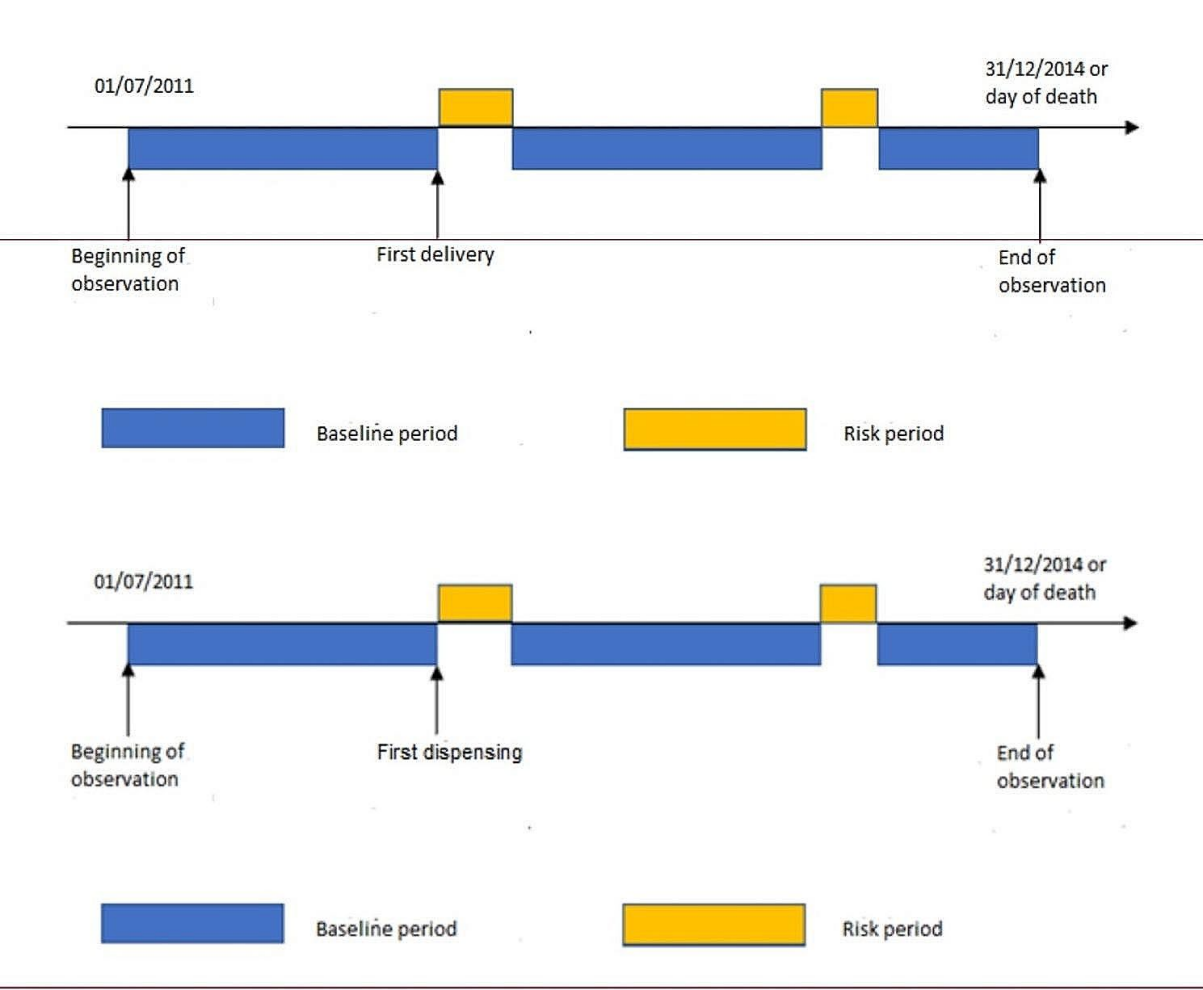
Study design of the self controlled case series. All subjects with an event of interest (first hospitalisation for an ischemic event) and exposed to triptans before the event were selected (from the exposed cohort). They were observed from the beginning of the cohort (01/07/2011) or when aged 65, to the end (31/12/2014). The rate of the event of interest was compared between risk periods (yellow) following triptan exposure (number of days in DDD plus 10 days) and baseline periods (dark blue)

This analysis estimates the relative risk of the event of interest in risk periods compared with all other periods (baseline periods). Patients with at least one event of interest were selected in in the observation period, i.e. when they were aged 65 years old or from on 01/07/2011 to 31/12/2014 or on the date of death (Fig. 2).

For each subject, the “risk period” began the day of triptan dispensing and ended ten days after the last day of exposure. This period was added to ensure that triptans have been completely eliminated from the body, since the ½ life of all triptans and their active metabolites (except flovatriptan) ranges between 2 and 5 h, leading to a complete elimination between half a day and 36 h (flovatriptan with ½ life of 24 h may be eliminated in 7 days) To ensure an important assumption of SCCS that recurrent events in the same subject are independent, if an ischemic event (any) was followed by other events within the 6 months after the first event we restricted the analysis to the first event, both for the risk periods and the baseline periods.

#### Potential confounders

In addition to the covariates used in the cohort study, additional potential confounders were considered for CCO and assessed within the risk period and the control periods: exposure to monoamine oxidase inhibitors (MAOIs), selective serotonin reuptake inhibitors (SSRIs), non-steroidal anti-inflammatory drugs (NSAIDs), opioids, antiplatelet drugs, drugs for the cardiovascular system, caffeine-containing drugs, exposure to levothyroxine, and season of event occurrence (spring (as reference): March to May, summer: June to August, autumn: September to November and winter: December to February).

#### Statistical analysis

##### For the primary analysis: propensity score matched cohort study

Descriptive statistics were used to describe the characteristics of the cohort, cases and matched controls. Continuous variables were described as mean and standard deviation (SD), qualitative variables were summarized using frequencies and percentages.

A Cox proportional hazard regression model, with stratification on matched pairs, was used to investigate the association between triptan exposure and outcomes. Univariate analyses were first performed to select the variables with a P value < 0.20, followed by a multivariate approach using backward selection and P value < 0.05 for statistical significance. Relevant interactions between covariates were checked. Proportional hazard assumption was tested for all covariates using interaction with time. The crude and adjusted hazard ratios (HRs) and their 95% confidence intervals (95% CI) were estimated.

Complementary analyses were performed on triptan exposure modelling, using cumulative triptan exposure since index date and considering triptan exposure as a time-varying variable. Sensitivity analyses were performed because the uncertainty related to definition of triptan exposure by adding 7, 14 and 28 days to the triptan duration (in DDD) and considering 7, 14 and 28 after the day of dispensing instead of exposure in number of DDD.

##### For the secondary analysis: CCO and SCCS

A conditional logistic regression model was used to estimate odds ratios (ORs) and their corresponding 95% CI in the CCO analysis, adjusted on the predefined covariates. A control-crossover analysis was performed to take into account the temporal trend of triptan use. This control sample included event-free triptan users matched with cases by age, sex and Charlson score (1:1). Index date (date of matching), case and control periods were the same as those in the CCO. This method estimated the odds ratio of the triptan-dispensing trend from 2011 to 2014.

In the SCCS analysis, the relative risk was estimated using a Poisson regression model, through the standard and pseudo-likelihood methods. The standard method took into account all exposure periods and events occurring during these periods. The pseudo-likelihood method took into account only the exposure periods before the event. Subsequent exposure periods were redefined as part of the baseline period and the number of events occurring during those exposure periods was adjusted to the number of events occurring in the baseline period.

The events of interest were analyzed simultaneously and separately (cardiac and cerebral events), to investigate whether the effect of triptans on receptors 5HT1B/D in different arteries may differ.

Sensitivity analyses were performed, by adding 7, 14 and 28 days to the period of exposure in the CCO and by excluding patients exposed to opioids in the SCCS All statistical analyses were performed using SAS 9.4® (SAS® Institute Inc, Cary NC, USA).

### Data protection and ethics

This study protocol was approved and received all mandatory authorizations according to the French regulations (*Institut des Données de Santé* approval in June 2014 and *Commission Nationale de l’Informatique et des Libertés* authorization in December 2014). Given that data are anonymous, no informed consent was required for studies based on French health insurance databases. This study, called TRUE for « Triptan Use and serious vascular events elderly over 65 years », was registered in the post-authorization survey registry of the European Network Centers for pharmacoepidemioly and Pharmacovigilance (ENCePP) coordinated by the European Medicine Agency (EUPAS n°8976).

## Results

### Primary analysis: cohort study

#### Characteristics of the population

The initial cohort included 47 353 incident users of triptans and 189 412 controls. Mean (SD) age was 71 years (6.0), with 71% of women. Table 1 shows the demographic and clinical characteristics of triptan users and controls, before and after propensity score matching. In unmatched comparisons, the control group tended to exhibit more comorbidities than the triptan group: Charlson’s score (65.3% vs. 75.9% without comorbidity), hypertension (63.8% vs. 53.9%), diabetes (20.4% vs. 11.9%), cardiovascular disease (39.2% vs.25.7%), and dyslipidemia (43.9% vs. 37.1%). After propensity score matching, the matched cohorts were well-balanced in terms of all observed covariates (Table 1). The propensity score-matched cohort included 24 774 triptan users and 99 096 matched controls. Mean (SD) age was 71 years (5.9) and 74% were women.

**Table 1:** Baseline demographic and medical characteristics for the triptans and control groups before and after propensity score matching

	Before propensity score matching		After propensity score matching	
	Triptan group (*n* = 47 353)	Control group (*n* = 189 412)	Triptan group (*n* = 24 774)	Control group (*n* = 99 096)
**Baseline characteristics**				
Female sex, No.(%)	33,809 (71.4)	135,236 (71.4)	18,367 (74.1)	72,996 (73.7)
Age, mean (SD)	71.7 (6)	71.7 (6)	71.6 (6)	71.5 (6)
Comorbididity, No.(%)				
Hypertension	25,540 (53.9)	120,848 (63.8)	18,495 (74.5)	74,296 (75)
Cardiovascular diseases	12,148 (25.7)	74,334 (39.2)	11,818 (47.7)	47,629 (48.1)
Myocardial infarction	393 (0.83)	3557 (1.88)	393 (1.59)	1912 (1.93)
Cardiac failure	600 (1.27)	5392 (2.85)	624 (2.52)	2672 (2.70)
Peripheral disease	893 (1.88)	6939 (3.66)	857 (3.45)	3703 (3.74)
Cerebrovascular disease	994 (2.1))	6832 (3.61)	897 (3.62)	3688 (3.72)
Dyslipidemia	17,561 (37.1)	83,076 (43.9)	12,314 (49.7)	49,077 (49.5)
Diabetes	5616 (11.9)	38,638 (20.4)	5764 (23.3)	23,191 (23.4)
**Charlson comorbidity index No.(%)**				
0	35,934 (75.9)	124,232 (65.5)	14,645 (59.1)	58,427 (59)
≤ 2	9798 (20.7)	50,964 (26.9)	8453 (34.1)	34,160 (34.5)
3–4	1235 (2.6)	9557 (5.1)	1259 (5.1)	4970 (5)
≥ 5	386 (0.8)	4659 (2.5)	417 (1.7)	1539 (1.6)
Number of hospitalisations, days, mean (SD)	0.9 (5)	2.4 (9.8)	1.7 (7)	2.1 (9)
Number of medical visits, mean (SD)	5.5 (5.0)	6.0 (5.2)	6.5 (5.2)	6.3 (4.9)

#### Main outcome: ischemic vascular events at 90 days

Within the 90 days after index date, 163 cardiovascular events were observed in the triptan group (0.66%) vs. 523 in the unexposed group (0.53%). The typology of vascular events observed differed between groups, with more acute cerebral events in the triptan group (Table 2).

**Table 2:** Detail of vascular events in the 90 days of exposure for the triptans and control groups

	Triptan group (*n* = 24 774)	Control group (*n* = 99 096)	P value
Number of vascular events, n (%)	163 (0.66)	523 (0.53)	
Acute cardiac events, n (%)	75 (0.30)	240 (0.24)	0.43
Angina pectoris	45	171	
Acute myocardial infarction	20	52	
Other acute ischemic heart diseases	10	17	
**Acute cerebral events, n (%)**	**68 (0.27)**	**146 (0.15)**	**0.0009**
Cerebral infarction	51	101	
Occlusion and stenosis of precerebral arteries, not resulting in cerebral infarction	12	37	
Stroke, not specified as haemorrhage or infarction	5	8	
**Other vascular events, n (%)**	**20 (0.08)**	**137 (0.13)**	**0.0002**
Arterial embolism and thrombosis	17	121	
Other retinal vascular occlusions	1	3	
Acute vascular disorders of intestine	1	9	
Other peripheral vascular diseases	1	4	

In the Cox regression model, triptan exposure was associated with ischemic vascular event (adjusted hazard ratio (aHR) HR 1.25; 95% Confidence Interval [1.05–1.49] (Table 3)). Risk estimates were varying with alternative exposure modelling: in the time-varying analysis, being “currently exposed at the time of event” was associated an HR of 8.74 [95% CI, 5.21–14.66]. Accumulated doses since index date were associated only for the lowest range of cumulative doses (1.85 [1.42–2.41]). Sensitivity analyses by modifying the definition of triptan exposure found similar results (supplementary Table 3).

**Table 3:** Association between triptan exposure and vascular events at 90 days (Cohort study, Cox model)

	Hazard ratio (95%CI)	Adjusted hazard ratio (95% CI) Exposed-unexposed	Adjusted hazard ratio (95% CI) Cumulative dose	Adjusted hazard ratio (95% CI) Time-varying
**Exposure to triptans**				
Controls (non-exposed)	1	1		
Exposure to triptans (Triptan group)	1.243 (1.043–1.483)	1.252 (1.050–1.494)		
**Exposure to triptans (time-varying)**				
Currently exposed at the time of event	8.691 (5.186–14.564)	-	-	8.745 (5.214–14.668)
Not currently exposed at the time of event	0.930 (0.760–1.137)			0.926 (0.757–1.133
**Cumulative dose (triptans) since index date (DDDs)**				
Controls (non-exposed)	1	-	1	-
< 12	1.861 (1.431–2.420)		1.852 (1.424–2.410)	
12	1.172 (0.876–1.566)		1.173 (0.877–1.569)	
> 12	0.600 (0.383–0.940)		0.598 (0.382–0.937)	
**Charlson’s score (0)**	1			
1–2	0.949 (0.644–1.399)	-	-	-
3–4	1.152 (0.674–1.967)			
> 5	1.337 (0.646–2.767)			
**Days of hospitalization (0)**	1			-
≤ 4	0.861 (0.539–1.375)	-		
> 5	0.852 (0.537–1.351)			
**Number of medical visits (0–2)**	1	-	-	-
3–4	1.154 (0.667–1.996)			
5–7	0.907 (0.537–1.531)			
> 7	1.223 (0.759–1.971)			

#### Secondary outcomes: all-cause death and death due to vascular events

All-cause death at 90 days was more frequent in controls than in the triptan group (*n* = 610, 0.63% versus *n* = 114, 0.46%, *p* = 0.00002). Death in the 30 days after being hospitalized for an ischemic cardiovascular event (90 days after index date) was also more frequent in controls than in the triptan group (*n* = 196; 0.20% versus (*n* = 26; 0.10%, *p* = 0.0005). Due to the very low number of events, no further analysis was carried out.

### Secondary analysis

#### Case-crossover study

Two hundred and nine patients corresponding to discordant pairs were included in the case-crossover analysis. Triptan exposure was significantly associated with all ischemic events in the final model adjusted for opioids, antiplatelets, number of days of hospitalization and season (adjusted OR, 1.63 [, 1.22–2.19]), (Table 4).

In the analysis stratified by the type of event, we found a relationship between cerebral events and triptan exposure but not for cardiac events. The result of the sensitivity analyses (supplementary Table 4) were consistent with that of the primary analysis. The trend of triptan use over the study period examined through the control-crossover design did not find any change in the use of these drugs (OR: 1.12 [0.50–2.51).

**Table 4:** Odds Ratios associated with vascular events (case-crossover study, conditional logistic regression model)

	Population	Adjusted Odds ratio (95% CI)
**All events**	209	
**Exposure to triptans**		1.63 (1.22–2.19)
**Covariates**		
Exposure to opioids		2.08 (1.21–3.59)
Exposure to antiplatelet drugs		3.38 (1.65–6.93)
Number of days of hospitalisation		1.19 (1.02–1.38)
Season		
Summer vs. spring		*0.83 (0.52–1.32)*
Automn vs. spring		*1.03 (0.64–1.66)*
Winter vs. spring		*1.08 (0.69–1.69)*
**Cerebral events**	**75**	2.14 (1.26–3.63)
**Cardiac events**	**89**	1.41 (0.91–2.20)

#### Self controlled case series

In total, 1804 patients were included in the SCCS, with a mean duration of the observation period of 3.42 years and an average duration of triptan exposure of 44.15 days. The relative incidence of all ischemic events was 2.13 [1.76–2.58] according to the standard method, and was 3.53 [2.89–4.31] according to the pseudo-likelihood method.

Associations remained statistically significant for cardiac (1.67 [1.23–2.27]) and cerebral events (3.20 [2.30–4.45]). Sensitivity analysis excluding patients exposed to opioids found similar results (RI all events 1.92 [1.27–2.90] in standard analysis; 3.53 [2.89–4.31 in pseudo-likelihood analysis).

## Discussion

To our knowledge, this is the first pharmacoepidemiological study investigating the association between triptan use among the elderly and hospitalization for acute vascular events. We found that triptan use was associated with a low increased risk for vascular events around the date of the first initiation, with a risk period corresponding to current exposure. Triptan users have less comorbidity, including cardiovascular comordities than the control group. In addition, the incidence of cardiovascular outcomes was low among triptan users. Due to careful attention of physicians prescribing triptans, it is possible that patients with cardiovascular pathology had contraindications for triptans which may explain the low incidence of cardiovascular outcomes among triptan users.

Few studies have assessed the relationship between triptans and vascular events [1–4, 11, 29]. Velentgas et al. [7], investigated the rates of vascular events in relation to the dispensing of triptans and ergotamine among a sample of US migraineurs between 1995 and 1999. They found an incidence of 1.4/1000 person-years for myocardial infarction but no increased risk associated with exposure to triptans or ergot alkaloids. Hall et al. [5] investigated, using the CPRD, the incidence of stroke, cardiovascular events and death in a cohort of British migraine patients included between 1992 and 1999 and followed 3 years, and found that, in general practice, triptan treatment did not increase the risk of these events. Albieri et al. studied the risk of stroke in migraine patients using triptans in Denmark [31]. They concludes that this risk is slightly higher than in the general population, but emphasize that the risk found is however very low and very close to being insignificant (1.07, 95% CI [1.01–1.14])

In a regional pharmacoepidemiological study conducted on the French health insurance database, Lugardon et al. also concluded that there was no cardiovascular impact in relation with overuse of triptans [32]. However, the authors underlined the lack of statistical power of their study. Finally, a disproportionality study conducted by Roberto et al. based on pharmacovigilance data from the FDA [33] has showed that three types of adverse events (ischemic strokes, arterial aneurysms and dissections, and vascular events related to pregnancy) were reported more frequently as suspected adverse drug reaction to triptans than to any other drug in the database. The authors concluded that there was a need to confirm the causal relationship between triptans and the above mentioned adverse events with large-scale clinical studies, and stressed the importance of a cardiological assessment before initiating treatment with triptans. Wannes-van der Heijden et al. [8] explored the overuse of triptans and ergotamine and the risk of vasoconstrictive complications in a Dutch population of triptan and triptan and ergotamine users. The overuse of triptans (defined by more than 90 DDDs per year) did not increase the risk of cerebral, cardiovascular or peripheral ischemic complications in either the general population or the population using cardiovascular drugs.

Recently Petersen et al. have studied whether an association between triptan and ischemic event exist, using a case-crossover design on the nationawide Danish registries [34]. Among the 429 612 subjects with a first-ever prescription of a triptan, 11 (0.003%) redeemed this first-even within one of the focal or referent windows preceding an acute myocardial infraction, 18 (0.004%) had ischemic stroke. The case-crossover analyses for the outcome myocardial infarction showed an OR estimated to 3.3 [CI 95% 1-10.9] and for the ischemic stroke an OR estimated 3.2 [CI 95% 1.3–8.1].

In spite of our choice of an at-risk population of elderly, our findings are consistent with that of those previous studies, showing that the increase in the relative risk of vascular events, when observed, is quite moderate.

One of the main strengths of our study relies in the large health insurance database, which captures, independently of the socio-economic status, all the triptans prescribed and dispensed, all available asdispensing-only medicines in France, leading to identify and follow more than 45 000 older users of triptans. Furthermore, the personal identification number allowed the linkage to the hospitalization database, enabling us to identify precisely acute vascular events by using previously used and validated ICD codes. The large older population identified in this study is concordant with epidemiological studies showing that older patients could represent 5–8% of the population of triptan users [12, 36]. Although migraine is less prevalent in older than in younger age groups, the absolute increase in the total number of older age groups may lead to an increase in the total number of migraine patients [3, 37]. Consequently, more elderly migraine patients may seek medical attention.

The link between migraine and cardiovascular disease has been described in multiple meta-analyses. These studies show robust evidence of migraine association with stroke, obstructive coronary artery disease and myocardial infarction, cardiovascular mortality, and atrial fibrillation. People with migraine with aura have an increased risk of atrial fibrillation, myocardial infarction, and cardiovascular death compared with those without migraine. The exact mechanisms underlying the association between migraine and cardio- and cerebro-vascular events are still not well understood. [38–39]

Interestingly, one important result of our study is related to the less prevalent cardiovascular risk factors in triptan users, compared to controls, also pointing the careful attention of physicians prescribing triptan, as also suggested by others [1, 40, 41]. This selection bias was, at least partially, controlled in our study through the use of a propensity score (study groups are well balanced in terms of cardiovascular history). The large size of the triptan population allowed us to detect the low incidence of serious acute vascular events (less than 1%) and to split them into cerebral or cardiac localization. Interestingly, whatever the analysis, the cerebral events (stroke) were more prevalent among triptan users. In the case crossover analysis, the risk remained significant only for cerebral events. The existence of a link between migraine and stroke has been long investigated and confirmed in migraine with aura, particularly in young women [42–44]. The older population included in our study and the case crossover analysis allows us to exclude the proper effect of migraine, and to discuss the role of triptans in these events. Moreover, this association between acute cerebral events and triptans should not be considered as a simple reflect of a selection bias toward exclusion (by the prescribers) of triptan users at high risk of cardiovascular outcomes, as the bias appeared to be controlled in the analysis. Indeed, this trend for cerebral rather than cardiac localization of ischemic events is biologically plausible and could be explained the pharmacodynamics properties of triptans. From a pathophysiological point of view, triptans activate 5-HT1B and 5-HT1D receptors, impairing both cerebral vessels and, interestingly to a much lesser extent, coronary arteries and resulting in an important vasoconstriction [45, 46]. The over-representation of cerebral events can thus be interpreted as a direct consequence of the effect of these drugs, due to their cranioselectivity on 5HT1D receptors, which are more prevalent in the brain than in coronary arteries. Vascular events with cardiac localization (essentially represented by angina pectoris and acute myocardial infarction) would be more suggestive of an additive effect of triptans on previously pathological coronary arteries, through a trigger effect of the triptans [47].

In our study, the risk estimates for vascular events with cardiac localization in the stratified analysis were not significant, but this result should be confirmed by further studies due to the lack of power for this analysis (89 subjects in this group). In addition with triptans, exposure to opioids and antiplatelet drugs also increased the risk of ischemic events. Opioid-induced tachycardia, which can cause myocardial infarction, may explain this association. In addition, since the majority of opioids are represented by tramadol, the action on serotonin should also be taken into account. These results are consistent with those of the studies by Li et al. and Ray et al., noting that the use of opioids in non-cancer pain increases the risk of acute myocardial infarction and cardiovascular death [48–50].

The investigation of a potential dose-effect relation shows that increased risk was observed only for lowest doses (i.e. < 12 DDD, or only one dispensing) compared to cumulatuve exposure above 12 DDDs; and even with a significant lower risk for the hightest doses. This may reflect the short time to occurrence of the vascular events. Indeed, the events tend to occur early in the exposure and are very likely to result in a triptan therapy discontinuation, leading to classify these patients into low users. Conversely, important users are less likely to present an event, leading to this reverse association.

This study has some limitations. Like all studies that use healthcare databases, exposure to treatment is derived from drug-dispensing data instead of effective drug intake by the patient leading us to perform several sensitivity analyses. Another weakness of the study was the lack of information on migraine leading to use triptandispensing as a proxy for migraine. However, as triptan are licensed only for migraine (and cluster headache as described above) and require a prescription by a physician, we considered that it is likely that our population would represent a population of migraineurs. The third limitation concerns the lack of data on other cardiovascular risk, such as cigarette smoking, alcohol consumption, low physical activity, cerebral atherosclerosis, atrial fibrillation, overweight and obesity, syndrome of sleep apnoea, family history of vascular diseases. However, given the self-matched design characterizing the case crossover analysis and the short observation period, we may reasonably assume that such confounding risk factor were the same for intra-individual comparisons. However, these designs are likely to exacerbate the protopathic bias for which triptans could be inadvertedly taken for an headache that represents a prodrome of stroke.

And finally, due to our definition of the incident users, we cannot exclude a residual misclassification of prevalent users that use triptans sporadicly.

Migraine treatment in the older population requires careful consideration of increased medical comorbidities. Unfortunately, for most migraine drugs, both for acute and preventive treatment, efficacy studies are lacking for patients ≥ 65 years.

Newly introduced acute treatments include three small-molecule calcitonin gene-related peptide receptor antagonists (ubrogepant, rimegepant, zavegepant) and a serotonin (5-HT1F) agonist (lasmiditan). The advantages of the novel therapies include their efficacy, favorable side-effect profile, particularly in patients with atherosclerotic disease, as well as their tolerability [50].

Interestingly, most trials involving gepants did not pose higher age limits, or the upper limit was not younger than 75 years old. This will provide a pool of data on a large sample of elderly patients. Once the tolerance of gepants over 65 has been demonstrated, it would be interesting to do a head-to-head study of triptan and gepant over 65, with and without atherosclerotic disease.

## Conclusions

To the best of our knowledge, this is the first study investigating the risk of vascular events among incident triptan users aged more than 65 years. The incidence of vascular is low among older triptan user. We found that triptan use among older may be associated with a low increased risk for serious vascular events.

### Electronic supplementary material

Below is the link to the electronic supplementary material.

Supplementary Material 1

## Data Availability

Study protocol: Our approach is fully described in the main text and in the Supplement. According to data protection and the French regulation, we cannot publicly release the data from the SNDS. However, any person or structure, public or private, for-profit or nonprofit, is able to access SNDS data upon authorization from the French Data Protection Office in order to carry out a study, research, or an evaluation of public interest (www.snds.gouv.fr/SNDS/Processus-d-acces-aux-donnees).
