# Supplementary material for: Triptan use in elderly over 65 years and the risk of hospitalization for serious vascular events
Source: J Headache Pain. 2024 Apr 26;25(1):68. doi: 10.1186/s10194-024-01770-x (PMC11055320; doi:10.1186/s10194-024-01770-x)
Supplement: Supplementary file 1 — Supplementary Material 1 [file 10194_2024_1770_MOESM1_ESM.docx]

# SUPPLEMENTARY CONTENT

Supplementary table 1. List of codes used for identifying covariates of interest

| **Covariate** | **Codes used for identification** |
| --- | --- |
| Hypertension | At least one dispensing within the framework of the long-term condition scheme (LTD) for hypertension  OR at least one dispensing of antihypertensive drug (ATC code C02) |
| Cardiovascular disease | At least one dispensing within the the long-term condition scheme (LTD) for cardiovascular diseases  1: Disabling stroke,  3: Chronic arteriopathies with ischemic manifestations,  5: Severe heart failure, arrhythmias, valvular cardiomyopathy, congenital cardiomyopathy  13: Coronary heart disease |
| Dyslipidemia | - Hospitalisation associated with an ICD-10 diagnosis code for dyslipidemia (E78.0, E78.1, E78.2, E78.3, E78.4, E78.5) at any diagnostic position (main, related, or associated diagnosis)  Or at least one dispensing of lipid modifying agents (ATC C10) |
| Diabetes | Hospitalisation associated with an ICD-10 diagnosis code for diabetes (E10-E14)  Or at least one dispensing of antidiabetic drug or insulin (ATC A10B, A10A).  Or at least one dispensing within the long term disease scheme for diabetes (LTD number 8) |
| Charlson comorbidity index | Algorithms defined by Bannay et al. |
| Exposure to Monoamine oxidase inhibitors (MAOIs) | At least one dispensing of monoamine oxidase inhibitors (ATC code N06AF, N06AG) |
| Exposure to Selective serotonin reuptake inhibitors (SSRIs) | At least one dispensing of selective serotonin reuptake inhibitors (ATC code N06AB) |
| Exposure to non-steroidal anti-inflammatory drugs (NSAIDs) | At least one dispensing of non-steroidal anti-inflammatory drugs (ATC code M01A) |
| Exposure to Opioids | At least one dispensing of opioids (ATC code N02A) |
| Exposure to antiplatelet drugs | At least one dispensing of antiplatelet agent ATC code B01A |
| Exposure to drugs for the cardiovascular system (chronic use only) | At least one dispensing of drug for the cardiovascular system  (ATC code C01, C02, C03, C07, C08, C09, C10) |
| Exposure to caffeine-containing drugs | At least one dispensing of drugs containing caffeine (ATC code N02BE71, N06BC01) |
| Exposure to levothyroxine | At least one dispensing of levothyroxine (ATC code H03AA01) |
| Season of event occurence | Spring: March to May  summer: June to August  autumn: September to November  winter: December to February |

Supplementary table 2. List of ICD-10 codes used for identifying vascular events

|  |  | |
| --- | --- | --- |
| **Category** | **ICD-10 Code** | **ICD-10 code label** |
| Acute cardiac events | I20x | Angina pectoris |
|  | I21x | Acute myocardial infarction |
|  | I22x | Subsequent myocardial infarction |
|  | I23x | Certain current complication following acute myocardial infarction |
|  | I24x | Other acute ischemic heart diseases |
|  | I46x | Cardiac arrest |
| Acute cerebral events | I63x | Cerebral infarction |
|  | I64x | Stroke, not specified as hemorrhage or infarction |
|  | I65x | Occlusion and stenosis of precerebral arteries, not resulting in cerebral infarction |
|  | I66x | Occlusion and stenosis of cerebral arteries, not resulting in cerebral infarction |
|  |  |  |
| Other vascular events | I73x | Other peripheral vascular diseases |
|  | I74x | Atrial embolism and thrombosis |
|  | H34.0 | Transient retinal artery occlusion |
|  | H34.1 | Central retinal artery occlusion |
|  | H34.2 | Other retinal artery occlusion |
|  | H34.8 | Other retinal vascular occlusion |
|  | H34.9 | Retinal vascular occlusion, unspecified |
|  | N28.0 | Ischemia and infarction of kidney |
|  | K55.0 | Acute vascular disorders of intestine |

“x” stands for “all following codes” (e.g “I20x”=”I20.0 to I20.9”)

Supplementary table 3. Sensitivity analyses for Cox regression models for all vascular events in the propensity score-matched cohort, with modification of triptan exposure definition.

|  | **HR (95% CI)** |
| --- | --- |
| **Triptan exposure in number of supplied DDD (days)** |  |
| + 7 days | 6.824 (4.573-10.183) |
| + 14 days | 5.973 (4.176-8.544) |
| + 28 days | 5.853 (4.301-7.965) |
|  |  |
| **Triptan exposure from de first date of dispensing (days)** |  |
| + 7 days | 8.000 (4.111-15.568) |
| + 14 days | 7.000 (4.400-11.136) |
| + 28 days | 6.272 (4.384-8.974) |

Cox regression models with calculation of hazard ratios (HR) with their 95% confidence interval, with time-varying triptan exposure

Supplementary table 4. Crude and adjusted Odds ratio for vascular events with sensitivity analyses for the duration of risk period (Case-crossover study)

|  | **Crude OR**  **(95% CI)** | **Adjusted OR**  **(95% CI)** |
| --- | --- | --- |
| **All vascular events** |  |  |
| 10-days (risk period retained) | 1.58 (1.20-2.09) | 1.63 (1.22-2.19) |
| 7-day | 1.58 (1.19-2.09) | 1.57 (1.17-2.10) |
| 14-day | 1.60 (1.21-2.13) | 1.59 (1.18-2.14) |
| 28-day | 1.55 (1.16-2.07) | 1.56 (1.15-2.13) |

Adjusted OR: final conditional logistic regression model adjusted for number of days of hospitalization, opioid exposure, and anti-platelet aggregation exposure
